# Functionalization of Silica Nanoparticles to Improve Crosslinking Degree, Insulation Performance and Space Charge Characteristics of UV-initiated XLPE

**DOI:** 10.3390/molecules25173794

**Published:** 2020-08-20

**Authors:** Yu-Wei Fu, Yong-Qi Zhang, Wei-Feng Sun, Xuan Wang

**Affiliations:** Key Laboratory of Engineering Dielectrics and Its Application, Ministry of Education, School of Electrical and Electronic Engineering, Harbin University of Science and Technology, Harbin 150080, China; yuwei_fu@126.com (Y.-W.F.); kingstel@163.com (Y.-Q.Z.)

**Keywords:** crosslinked polyethylene, dielectric nanocomposites, photon-initiator, auxiliary crosslinker, space charge characteristics

## Abstract

In order to inhibit the outward-migrations of photo-initiator molecules in the ultraviolet-initiated crosslinking process and simultaneously improve the crosslinking degree and dielectric properties of crosslinked polyethylene (XLPE) materials, we have specifically developed surface-modified-SiO_2_/XLPE nanocomposites with the silica nanofillers that have been functionalized through chemical surface modifications. With the sulfur-containing silanes and 3-mercaptopropyl trimethoxy silane (MPTMS), the functional monomers of auxiliary crosslinker triallyl isocyanurate (TAIC) have been successfully grafted on the silica surface through thiol–ene click chemistry reactions. The grafted functional groups are verified by molecular characterizations of Fourier transform infrared spectra and nuclear magnetic resonance hydrogen spectra. Scanning electronic microscopy (SEM) indicates that the functionalized silica nanoparticles have been filled into polyethylene matrix with remarkably increased dispersivity compared with the neat silica nanoparticles. Under ultraviolet (UV) irradiation, the high efficient crosslinking reactions of polyethylene molecules are facilitated by the auxiliary crosslinkers that have been grafted onto the surfaces of silica nanofillers in polyethylene matrix. With the UV-initiated crosslinking technique, the crosslinking degree, insulation performance, and space charge characteristics of SiO_2_/XLPE nanocomposites are investigated in comparison with the XLPE material. Due to the combined effects of the high dispersion of nanofillers and the polar-groups of TAIC grafted on the surfaces of SiO_2_ nanofillers, the functionlized-SiO_2_/XLPE nanocomposite with an appropriate filling content represents the most preferable crosslinking degree with multiple improvements in the space charge characteristics and direct current dielectric breakdown strength. Simultaneously employing nanodielectric technology and functional-group surface modification, this study promises a modification strategy for developing XLPE nanocomposites with high mechanical and dielectric performances.

## 1. Introduction

Crosslinked polyethylene (XLPE) is a representative insulating material of thermo-setting homopolymers derived from polyethylene, which inherits the excellent electrical insulation performances of polyethylene and acquires a higher heat resistance and better physical-mechanical properties [1]. Ever since its inception in the 1960s, the XLPE development has made great progress in the field of power cable insulation, so that the voltage level of XLPE insulated cables has approached 500 kV in the early 21st century. The traditional technique of chemical crosslinking peroxides in the production of high-voltage XLPE insulated cables cannot avoid burning materials, which will form dielectric defects in insulation layers after long-term operations of manufacturing equipment. Therefore, it is impossible to achieve sufficiently long high-voltage cables through thermochemical crosslinking technologies. In recent years, ultraviolet (UV) crosslinking technology has attracted considerable interest due to its high production speed, low cost of raw materials, and minimal radiation damage to insulating materials. Related researches now primarily focus on the preparations of photon-initiation systems [2,3,4], the optimization of UV irradiation light sources [5,6] and the improvement of insulation performances of UV-initiated XLPE (UV-XLPE) materials [7,8]. As one of the most important components in UV-initiated crosslinking formulations, the effective photon-initiator should efficiently absorb UV irradiation in a suitable wavelength range and match the UV light emission spectra to obtain high quantum initiation yields. Photon-initiation systems with small molecular weights such as benzophenone (BP) and triallyl isocyanurate (TAIC) have been verified to be efficiently applied for polyethylene crosslinking reactions in the industrial production of XLPE cables [9,10]. However, the poor compatibility with polyethylene (PE) of the small photon-initiator and auxiliary-crosslinker molecules make them tend to migrate out of the polymeric blend even at ambient temperature, which will reduce the reaction rate and crosslinked degree of XLPE. Moreover, small molecular initiation systems with high volatility are prone to evaporate and deposit on the UV lamp cover in crosslinking process, leading to the abatement in UV light transmission efficiency and corrosion of irradiation equipment [7,8]. It is hereby foreseen that the development of a new photon-initiation system with low volatility and good compatibility with polyethylene molecules is the first task for developing UV-XLPE technology of manufacturing high-voltage cables.

The surface modification technology of nanodielectrics (polymer dielectric nanocomposites) suggests a strategic routine for improving the electrical, thermal and mechanical properties of cable insulating materials [11,12,13]. The introduction of inorganic nanoparticles can endow polymer nanocomposites with significant amelioration in insulation performances, such as the enhanced breakdown field strength [14], suppressed space charge accumulation [15] and improved electric-tree resistance [16], and will simultaneously improve the thermal conductivity, thermostability and mechanical properties such as tensile strength and impact strength [17,18]. Currently, the intensively studied inorganic nanofillers for modifying XLPE materials are primarily made from silica (SiO_2_) or magnesium oxide (MgO). Due to the extensive source, large specific surface area, high reactivity, non-toxic and pollution-free chemistry, and visible light transparency, the nano-scale SiO_2_ materials have been widely studied in the development of UV-cured nanocomposites [19]. Ever since Decker first proposed the idea of applying functionalized SiO_2_ nanoparticles to UV-cured materials [20], a series of UV-cured nanocomposites such as SiO_2_/acrylic polyurethane, SiO_2_/epoxy resin and SiO_2_/methacrylate have been comprehensively developed to improve the mechanical and electrical properties of polymers [21,22,23]. This is a valid strategy of exploiting the inorganic SiO_2_ nanoparticles in polyethylene crosslinking reactions to improve the insulation performances of UV-XLPE insulating materials. SiO_2_/UV-XLPE nanocomposites with the functionalized SiO_2_ nanofillers are essentially developed by combining the photon-initiated crosslinking technology of polymers and the surface modification schemes of nanodielectrics, which can render various novel features of crosslinking configuration and effectively improve dielectric properties of UV-XLPE insulation materials.

In the present paper, we specifically develop XLPE nanocomposites with the nanofillers functionalized by auxiliary crosslinkers to prevent the outward-migration and volatilization of small molecular photo-initiation system in UV-initiated crosslinking process and simultaneously improve the crosslinking degree, space charge characteristics and breakdown electric field of high-voltage UV-XLPE insulating materials. Based on the thiol–ene click chemistry and the surface chemical modification [24,25], the auxiliary crosslinking agent of triallyl isocyanurate (TAIC) can be grafted onto the surfaces of SiO_2_ nanoparticles through silane coupling agent (3-mercaptopropyl trimethoxy silane, MPTMS) to achieve auxiliary crosslinking function on the surfaces of SiO_2_ nanoparticles [26,27,28]. Accordingly, the modified hybrid nanoparticles of TAIC-s-SiO_2_ are prepared and filled into polyethylene matrix to participate together with macromolecular photon-initiators in polyethylene crosslinking reaction, through which the surface-modified SiO_2_ nanofillers are introduced into the crosslinking configuration of polyethylene matrix. This scheme can considerably promote the compatibility of auxiliary crosslinkers (TAIC) with polyethylene molecules and simultaneously increase the dispersivity of SiO_2_ nanofillers in polyethylene matrix. Under the catalysis of triethylamine, the Michael reactions of the mercapto-double-bonds represent high reactivity in mild reaction conditions. The solvent and triethylamine with a low boiling temperature are used so that the residual compounds can be completely removed by vacuum distillation without further purification. To this end, modified-SiO_2_/UV-XLPE nanodielectrics have been developed by UV irradiation cross-linking technology with linear low density polyethylene (LLDPE) as matrix. The crosslinking degree, space charge characteristics and dielectric breakdown strength of these SiO_2_/XLPE nanocomposites with surface-modified hybrid nanofillers are investigated by testing and analyzing the nuclear magnetic resonance, infrared spectrum, scanning electron microscopy, dielectric breakdown strength and space charge distribution.

## 2. Material Preparation and Testing

### 2.1. Material Preparations

**Source Materials**. The original materials used for preparing nanocomposites are listed as follows: LLDPE (DFDA 7042, melting point 135 °C, density 924 kg/m^3^, MFI 2.0 g/min, Petrochina Jilin Petrochemical Co. Ltd., Changchun, China) as the matrix material; 4-Hydroxyl benzophenone laurate (BPL, chemically pure, Harbin University of Science and Technology, Harbin, China) and triallyl isocyanurate (TAIC, chemically pure, Macklin Biochemical Technology Co. Ltd., Shanghai, China) as the macromolecular photon-initiation crosslinking system; pentaerythritol ester antioxidant (Irganox1010, Shanyi Plastics Co. Ltd., Dongguan, China); 3-merraptnpropylt rimethnxysilane (MPTMS, chemically pure, Jiangsu Heyuan Chemical Co. Ltd., Nanjing, China) as the silane coupling agent; and acryloyl chloride (AC, Shanghai Aladdin Biochemical Technology Co. Ltd., Shanghai, China); Nanosilica (20 nm), dichloromethane (DCM), triethylamine (TEA), anhydrous ethanol (EtOH), tetrahydrofuran (THF), and xylene (Tianjin Fuyu Fine Chemical Co. Ltd., Tianjin, China) are used for the surface modifications of the silica nanoparticles. All materials are used as received.

**Figure 1:**
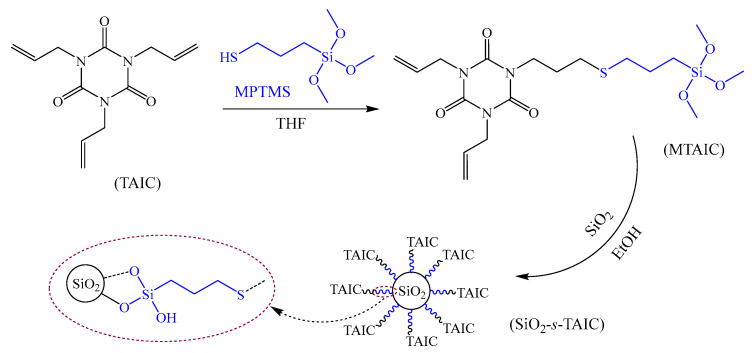
Reaction process of synthesizing crosslinker-functionalized nanosilica.

**Synthesis of Crosslinker-Functionalized Nanosilica**. The surface modification process of functionalizing SiO_2_ nanoparticles by grafting the auxiliary crosslinking agent is shown in Figure 1. The mixture solution of 3.92 g MPTMS (0.02 mol), 0.23 g TEA and THF is slowly dropped into the solution of 4.98 g TAIC (0.02 mol) dissolved in 10 mL THF solvent, stirred under nitrogen protection in ice-water bath for 5 min, and then naturally warmed to ambient temperature. The liquid product (MTAIC) with 76% yield will be obtained after reacting in thermal insulation for 24 h. By means of ultrasonic dispersion treatment for 30 min, 10 g dried nanosilica was uniformly blended into 100 mL ethanol solution (3:1), after which the MTAIC is instilled with the solution being controlled on pH = 4 by diluted hydrochloric acid. The obtained suspension is washed and filtered with ethanol for 3 times and then dried in vacuum at 60 °C to finally achieve the functionalized TAIC-*s*-SiO_2_ nanomaterial.

**SiO_2_/XLPE Nanocomposite Preparation**. According to the mixing ratios as listed in Table 1, four kinds of mixtures for preparing pure and composite UV-XLPE materials are obtained by the melt-blending machine and pressed into film samples by a plate vulcanizer. For photon-initiated crosslinking reactions, the blended film material is first treated in a plate vulcanizer at 160 °C with the pressure being increased by 5 MPa per 5 min from 0 to 15 MPa to make the material melt, and then irradiated by a light source array of UV LED units (NVSU233A-U365, Riya Electronics Chemistry Co. Ltd., Shanghai, China) for 2~5 min on an irradiation platform at normal pressure and room temperature in atmosphere [29]. In this pivotal process, exploiting UV-transparency of the LLDPE fluid at a temperature higher than the melting point after being squeezed out from the extruder, the UV lights are incident through the melting mixture. In the photon-initiated crosslinking process under UV irradiation, the power and wavelength of the light-emitting are controlled at 1.0 w and 365 nm respectively, and the light is incident along the direction at 60° angle with the plane of film sample. After short-circuit hot-degassing for 24 h at 80 °C in a vacuum oven to eliminate residual stresses and crosslinking by-products, three kinds of SiO_2_/XLPE nanocomposites are eventually prepared with the macromolecular photon-initiation crosslinking system (BPL/TAIC), as indicated by pure XLPE and the SiO_2_/XLPE nanocomposites with 0.5 wt%, 1.5 wt%, and 2.0 wt% TAIC-*s*-SiO_2_ nanofillers (0.5wt%TAIC-*s*-SiO_2_/XLPE, 1.5wt%TAIC-*s*-SiO_2_/XLPE, and 2.0wt%TAIC-*s*-SiO_2_/XLPE, respectively) in Table 1.

**Figure 2:**
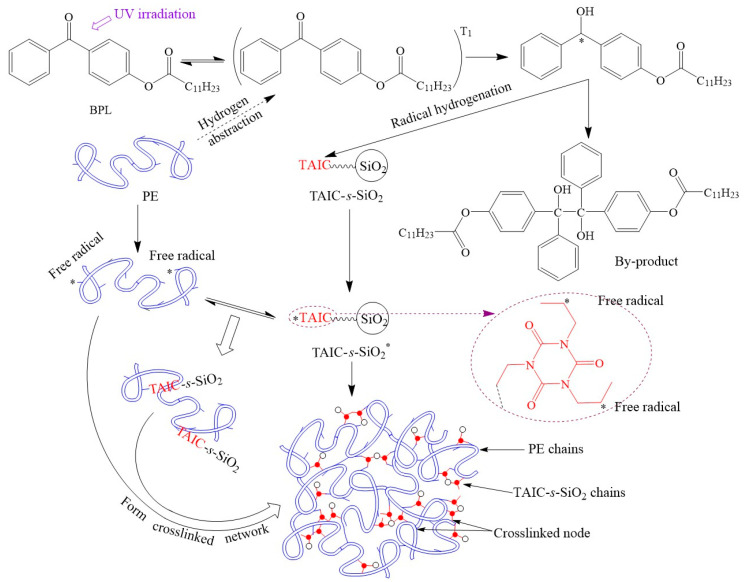
Schematic mechanism of polyethylene crosslinking reaction initiated by UV irradiation.

**Crosslinking Reaction Progress for SiO_2_/XLPE Nanocomposites**. In the UV-initiated crosslinking reaction for preparing SiO_2_/XLPE nanocomposites, as shown in Figure 2, the photon-initiator BPL absorbs the specific energy of UV photons to be excited from a single state to a triple state, which can abstract hydrogen from polyethylene (PE) molecule to form a chemical radical on PE molecule and become an active BPL with a carbonyl radical (BPL*) that may finally react each other to produce by-products [30]. Then, the allyl double bonds of TAIC on the modified surface of TAIC-*s*-SiO_2_ are hydrogenated by the active carbonyl groups of BPL* to generate allyl radicals. All of these radicals produced from the UV-initiation mechanism will finally couple to build a crosslinking network structure in which the TAIC-*s*-SiO_2_ nanoparticles have been dispersively introduced though TAIC in chemistry.

### 2.2. Material Characterization and Property Test

The content of hydrogen atoms (H) in the different groups of MTAIC molecules are determined by a nuclear magnetic resonance (Bruker-1, ^1^H NMR), in which the material structure can be characterized according to the type and quantity of H in the detected samples. The molecular structures of TAIC-*s*-SiO_2_ powder samples are further characterized by a Fourier transform infrared spectrometer (FTIR-6100, Jiasco Trading Co. Ltd., Shenyang, China) and the photon-initiated crosslinking efficiency for preparing UV-XLPE materials are analyzed by testing gel content. According to the standard of ASTM D 2765-01, gel content tests are carried out by the solvent extraction method. Gel contents of pure XLPE and SiO_2_/XLPE nanocomposites are calculated from the percentage of insoluble network crosslinked polymers.

**Figure 3:**
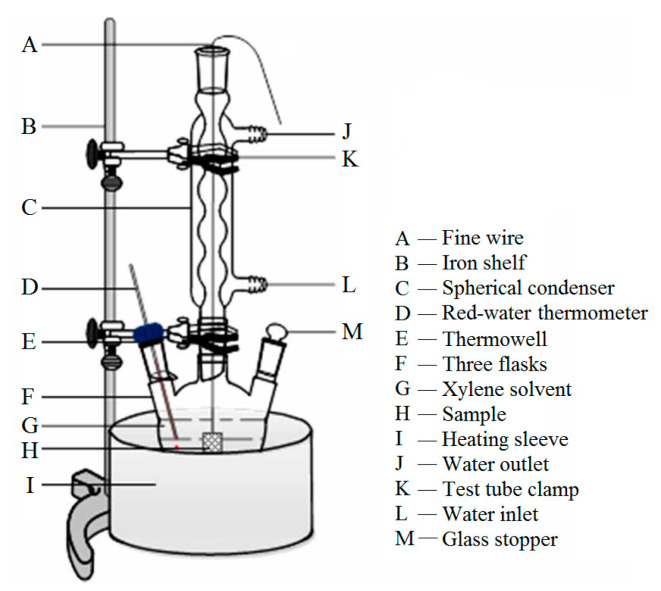
Schematic equipment of gel extraction experiment.

The gel extraction experiments are implemented using xylene as a solvent in the reaction container of three flasks, as shown in Figure 3. The thermometer at the side mouth of the flask is utilized to monitor the solvent temperature in real time so that the xylene solvent can be controlled in a slightly boiling state. The gel extraction experiment is specified as follows: (a) the material sample is weighed as M_1_ in mass and placed in a square (45 mm side length) mesh pocket made from a stainless steel filter screen, and then the total mass of the sample and mesh pocket is weighted as M_2_; (b) the stainless steel mesh is connected with aluminum wire and immersed into xylene solution; (c) the electronic heating device is operated to control the temperature at 130~140 °C so that the xylene solution has been heated to boiling and the condensate tube could drop about 20 water droplets per minute; (d) after 12 h, the stainless steel mesh pocket is taken out from the xylene solvent and placed into an oven at 80 °C for 12 h to completely evaporate the residual xylene solvent; and (e) finally, the stainless steel mesh pocket is removed and weighed as M_3_ in mass to calculate gel content by the equation (M_3_–M_2_)/M_1_.

In order to characterize the nanofiller distribution microstructures, scanning electron microscopy (SEM) characterizations are implemented to the brittle fractured cross-sections of samples that have been obtained by treating the prepared XLPE nanocomposites in liquid nitrogen and etching with n-heptane for 20 min. The cross-section SEM images are observed by an ultra-high-resolution cold field emission scanning electron microscope (SU8020, Hitachi Co. Ltd., Tokyo, Japan) with a magnification of 75 k under the accelerating voltage of 5~10 kV. Space charge characteristics of the prepared pure XLPE and SiO_2_/XLPE nanocomposites are measured with the pulsed electro-acoustic (PEA) system (HY-PEA-DPT01, HeYi Electric Co. Ltd., Shanghai, China) at various temperatures of 25~100 °C under direct current (DC) electric field of 40 kV/mm in polarization for 60 min, in which the tested materials are fabricated into 50 × 50 × 0.3 m^3^ film samples with both sides evaporated by the aluminum electrode films in 25 mm diameter. The DC dielectric breakdown strength (DBS) of the circular film samples with a diameter of 80 mm and a thickness of 1.0 mm are tested by asymmetric columnar electrodes (25 mm and 75 mm in diameter for high-voltage and ground electrodes, respectively) at the same testing temperatures of PEA. When the applied electric field is continuously increased at a constant speed of 4 kV/s, the possible maximum voltage is promptly recorded just before the samples have been broken down.

## 3. Results and Discussion

### 3.1. Material Characterization

**Figure 4:**
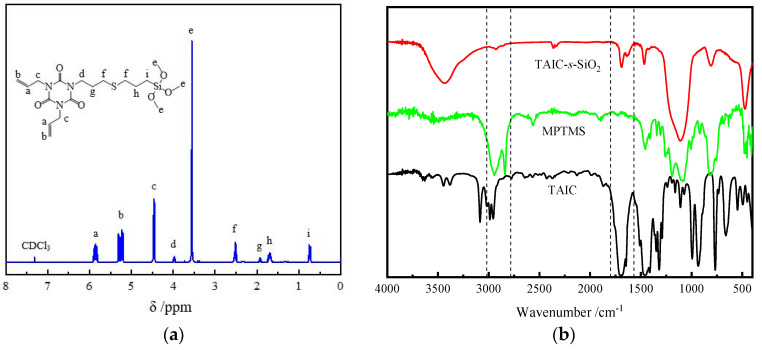
(**a**) ^1^H-NMR spectrum of MTAIC molecules and (**b**) FTIR transmission spectra of TAIC-*s*-SiO_2_, MPTMS and TAIC.

As illustrated by the hydrogen nuclear magnetic resonance (^1^H-NMR) spectrum of MTAIC molecules in Figure 4a, the measured peak positions of the synthesis products exactly coincide with theoretical results. The peak at *δ* = 5.85 ppm and 5.24 ppm identify the chemical displacement of H atom on –CH=CH_2_ and *δ* = 2.52 ppm indicates the H atom on –CH_2_– being adjacent to sulfur atoms (S), which demonstrate the successful synthesis of MTAIC. The prepared hybrid nanomaterial of TAIC-*s*-SiO_2_ that has been functionalized with auxiliary crosslinkers is characterized by the tested FTIR spectra as shown in Figure 4b. The characteristic infrared absorption peaks at 3438 cm^−1^, 2561 cm^−1^, 2956 cm^−1^, and 2841 cm^−1^ represent the intrinsic vibrations of Si–OH on the surface of SiO_2_ nanofillers, –SH in MPTMS molecules, methyl, and methylene, respectively. The characteristic peaks at 1645 cm^−1^ and 1690 cm^−1^ respectively derive from the double-bond stretching vibrations of –CH=CH_2_ and –C=O groups in TAIC molecules. It can be verified from the coordinately arising peaks of methyl/methylene (2915 cm^−1^), carbonyl (1689 cm^−1^), and ethylene (1637 cm^−1^) in the infrared spectrum of TAIC-*s*-SiO_2_ that TAIC molecules have been successfully grafted onto the surface of SiO_2_ nanoparticles. The FTIR and 1H-NMR tests also indicate that the grafting ratio of auxiliary crosslinking agents on the surfaces of TAIC-*s*-SiO_2_ nanoparticles could be modified by altering the amount of MTAIC in surface modification processes. It was reported that the C–O bond will break prior to the Si–C bond when the organically modified SiO_2_ nanoparticles being heated [31]. Therefore, the weight loss of the modified nanoparticles at a temperature lower than 250 °C originates from the thermal desorption of the physically adsorbed small molecules, while the weight loss at higher temperatures of 250~800 °C can be attributed to the decomposition of organic groups grafted on nanosurfaces. Taking into account that the functionalizing modification on the electrical properties of UV-XLPE nanocomposites can be ameliorated by altering the filling content of TAIC-*s*-SiO_2_ nanofillers, the samples with MTAIC dosage of 30 wt% are selected for preparing UV-XLPE nanocomposites filled with TAIC-*s*-SiO_2_ nanoparticles.

### 3.2. Crosslinking Degree of UV-XLPE Nanocomposites

**Figure 5:**
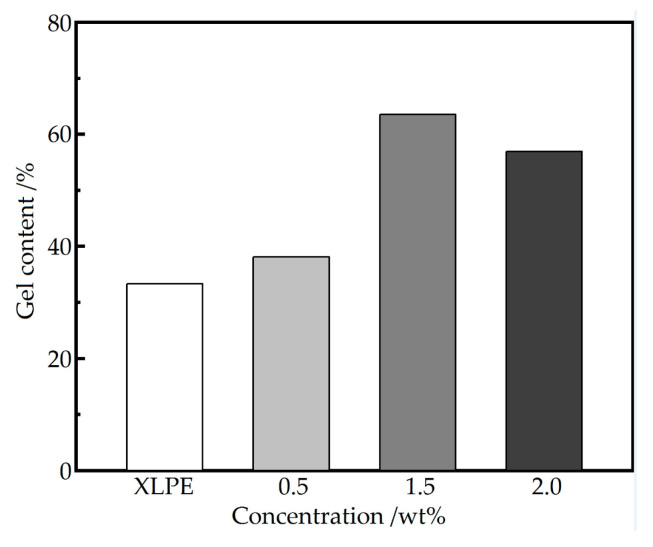
Gel contents of TAIC-*s*-SiO_2_/XLPE nanocomposites versus the filling concentration of Table 2. nanoparticles, through crosslinking reaction under UV irradiation for 300 s.

The irradiation time of 300 s is adopted in polyethylene crosslinking process for preparing the SiO_2_/XLPE nanocomposites to investigate the effect of TAIC-*s*-SiO_2_ concentration on crosslinking degree of polyethylene matrix, which can be estimated by gel content, as the tested results shown in Figure 5. With the filling content of TAIC-s-SiO_2_ being increased, the gel contents of SiO_2_/XLPE nanocomposites show the turning variation trends of first increasing and then decreasing. When the filling content of TAIC-*s*-SiO_2_ is no higher than 1.5 wt%, the TAIC-*s*-SiO_2_ nanofillers are well dispersed into a relatively smaller size with a larger specific surface area, which will bear more UV absorption at the interface between the TAIC-*s*-SiO_2_ and polyethylene matrix; when the TAIC-*s*-SiO_2_ concentration is increased to 2.0 wt%, the nanosilica aggregate into a larger size of particles with a remarkably reduced specific surface area in polyethylene matrix, which thereby abates the UV absorption and auxiliary crosslinking efficiency of the grafted TAIC on the surface of SiO_2_ nanofillers.

### 3.3. Morphology Characteristics of XLPE Nanocomposites

**Figure 6:**
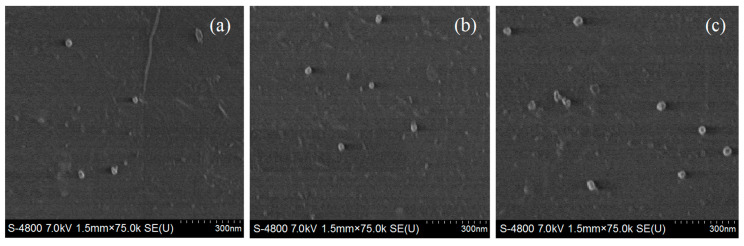
SEM cross-section images of (**a**) 0.5wt%TAIC-*s*-SiO_2_/XLPE, (**b**) 1.5wt%TAIC-*s*-SiO_2_/XLPE and (**c**) 2.0wt%TAIC-*s*-SiO_2_/XLPE nanocomposites.

The size and dispersion of SiO_2_ nanofillers in XLPE composites account for the correlated modification mechanism and accordingly determine the acquired dielectric properties of composite materials. TAIC-*s*-SiO_2_/XLPE nanocomposites are characterized by SEM, as illustrated in Figure 6. It is confirmed that the surface-modified TAIC-*s*-SiO_2_ nanofillers in XLPE composites are majorly distributed in the sizes of 20~60 nm with a preferable high dispersion, which substantially meet the requirements of our experimental proposition. The nanoparticle agglomeration in polyethylene matrix cannot be completely avoided for the composite materials prepared with the mechanical melting blend method. Nevertheless, SEM cross-section images indicate that the size of the surface-modified silica fillers remains in nanoscales even for 2.0 wt% filling content, as shown in Figure 6c, which means they will still play out the functions of nanofillers such as in successful nanodielectrics. For 0.5 wt% and 1.5 wt% filling contents, TAIC-*s*-SiO_2_ nanofillers with a slightly smaller size are uniformly dispersed over a larger distance without any appearance of particle agglomeration in comparison to the 2.0 wt% content.

### 3.4. Dielectric Breakdown Strength

Due to the statistic dispersion of breakdown electric fields measured for pure XLPE and all the composite samples, the 2-parameter Weibull statistics is implemented to evaluate DBS by fitting experimental results with the statistic distributions described as follows:

(1) $$F\left(E\right)=1-\exp\left[-{\left(\frac{E}{E_{b}}\right)}^{\beta }\right]$$

where *E* denotes the electric field for an electrical breakdown experiment; *E*_b_ represents the characteristic value of breakdown electric field with a probability of 63.2%; *β* signifies the shape parameter indicating the dispersivity of experimental data; and *F*(*E*) symbolizes the probability at a specific breakdown electric field *E*, which is expressed as following:

(2) $$F\left(E,n\right)\approx \frac{i-0.3}{n+0.4}\times 100\%$$

where *i* denotes the number in ascending order of the tested samples and *n* is the total number of the tested samples for the same material, which is equal to 10 for the present experiments.

**Figure 7:**
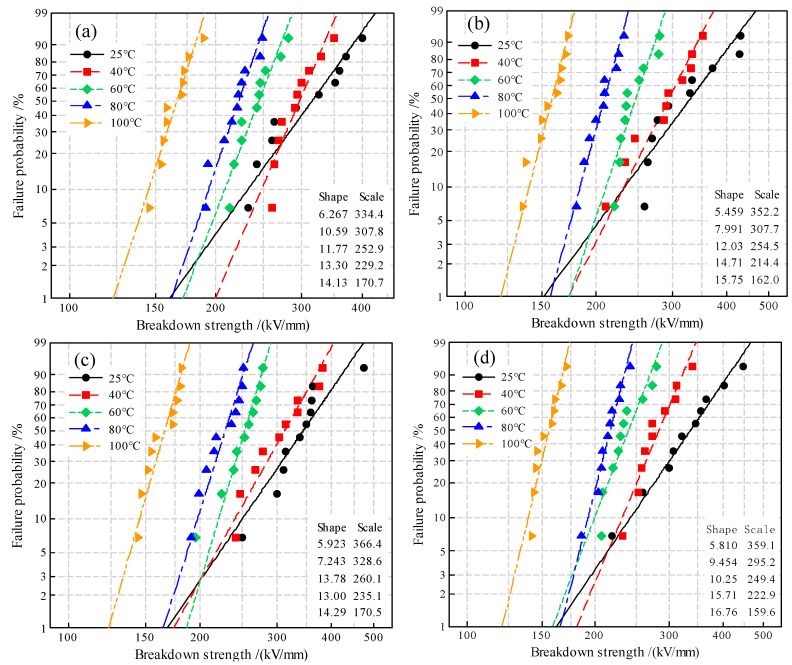
DBS statistics fitted with 2-parameter Weibull distribution at temperatures of 25~100 °C for (**a**) pure XLPE and the nanocomposites of (**b**) 0.5wt%TAIC-*s*-SiO_2_/XLPE, (**c**) 1.5wt%TAIC-*s*-SiO_2_/XLPE and (**d**) 2.0wt%TAIC-*s*-SiO_2_/XLPE.

The 2-parameter Weibull statistics is utilized to analyze the experimental results of the DBS tests in the temperature range of 25~100 °C, as shown in Figure 7. The scale parameter *E*_b_ indicates a characteristic breakdown electric field with a probability of 63.2%, and the shape parameter *β* represents the dispersion of breakdown data [32,33], as listed in Table 2. The shape parameters and characteristic breakdown fields of all the samples increase and decrease, respectively, as temperature rises, which means that the stability and strength of resisting dielectric breakdown will be degraded by atomic vibrations that are exacerbated when the temperature is raised. The breakdown strength of polymer materials has a great relationship with the molecular morphology, crystal structure, and material. With the increase in temperature, the average free path of electrons under the electric field increases due to the increase of free volume in polymer materials, leading to a higher electronic kinetic energy obtained from electrical acceleration, which will aggravate the electronic impacts on the chemical bonds of polymer molecules.

Weibull statistics of DBS tests show that the overall breakdown performance of 1.5wt%TAIC-*s*-SiO_2_/XLPE nanocomposite is better than the others in the investigated temperature range. The relatively lower breakdown field strength of 2.0wt%TAIC-*s*-SiO_2_/XLPE than that of 1.5wt%TAIC-*s*-SiO_2_/XLPE can be attributed to the reduced specific surface area of the aggregated silica nanofillers caused by excessively high filling content. In particular, 1.5wt%TAIC-*s*-SiO_2_/XLPE has achieved the most significant improvement in insulation stability at the temperatures of 60 °C, 80 °C and 100 °C, manifesting as the smallest shape parameter, which means the numerical distribution of DBS is the most concentrated. Meanwhile, the 1.5 wt% filling content is appreciably higher than the preferred content of filling neat SiO_2_ nanoparticles without surface modification. It has been normally recognized that preparing XLPE nanocomposites by filling neat SiO_2_ will engender substantial agglomerations of SiO_2_ nanofillers when the filling content is higher than about 1.5 wt%. Furthermore, TAIC molecules with polar-groups that have grafted on the surface of SiO_2_ nanoparticles will not be evaporated in the crosslinking reaction, thus retaining a higher density of polar-groups that act as effective charge traps to improve DBS by impeding carrier transports and inhibiting space charge accumulation through the trapping mechanism [34]. The 1.5wt%TAIC-*s*-SiO_2_/XLPE nanocomposite acquires a higher characteristic 63.2% DBS than the pure XLPE due to the deep traps introduced by the polar-groups of TAIC molecules grafted on nanosilica surfaces, which is consistent with the charge trapping and scattering mechanism in suppressing space charge accumulation and impeding electrical conductance under DC electric field. The TAIC-grafting surface modification of silica nanofillers for appropriate 1.5 wt% filling content retains a higher DBS even when the temperature increases to 80 °C, as shown in Table 2. Based on the elevated-temperature DBS results, it is reasonable to correlate DBS improvement with the increment of trap level depth and density. These results support the charge carrier trapping model and suggest that TAIC is an effective graft candidate to improve the insulation performances of polymer materials.

### 3.5. Space Charge Characteristics

**Figure 8:**
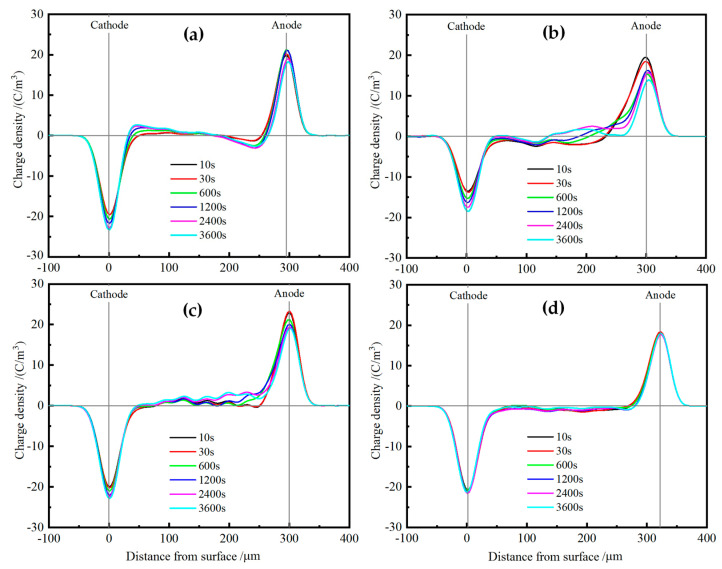
Space charge distributions in (**a**) pure XLPE and the nanocomposites of (**b**) 0.5wt%TAIC-*s*-SiO_2_/XLPE, (**c**) 1.5 wt% TAIC-*s*-SiO_2_/XLPE and (**d**) 2.0 wt% TAIC-*s*-SiO_2_/XLPE under DC electric field of 40 kV/mm at 25 °C.

Space charge distributions of XLPE and TAIC-*s*-SiO_2_/XLPE nanocomposites are tested under DC electric field of 40 kV/mm for 60 min at various temperatures from 25 °C to 80 °C by means of the pulsed electro-acoustic (PEA) method to elucidate the underlying physics of charge injections. Figure 8 illustrates the space charge accumulations at a temperature of 25 °C. The heterocharges evidently accumulate near both the cathode and anode in pure XLPE with the space charge density increasing to the highest peak value of 3 C/m^3^, and thus will exacerbate field distortion due to impurity ionization, as shown in Figure 8a. In contrast, no obvious charge injection can been found near both electrodes in TAIC-*s*-SiO_2_/XLPE nanocomposites even after polarizing for 60 min, and only a small amount of positive charges accumulate uniformly inside the 0.5wt%TAIC-*s*-SiO_2_/XLPE and 1.5wt%TAIC-*s*-SiO_2_/XLPE nanocomposites, as shown in Figure 8b,c. In particular, space charge accumulations have been suppressed throughout 2.0wt%TAIC-*s*-SiO_2_/XLPE nanocomposite, as shown in Figure 8d.

**Figure 9:**
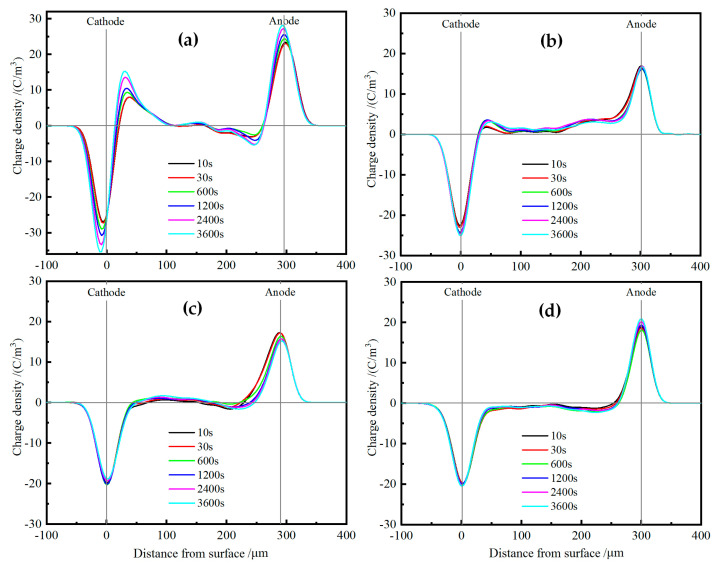
Space charge distributions in (**a**) pure XLPE and the nanocomposites of (**b**) 0.5wt%TAIC-*s*-SiO_2_/XLPE, (**c**) 1.5wt%TAIC-*s*-SiO_2_/XLPE and (**d**) 2.0wt%TAIC-*s*-SiO_2_/XLPE under DC electric field of 40 kV/mm at 40 °C.

When the testing temperature is increased to 40 °C, as shown in Figure 9, a large number of heterocharges accumulate near both electrodes and the distributed positions shift inward in pure XLPE as the polarization time prolongs. For the 0.5wt%TAIC-*s*-SiO_2_/XLPE nanocomposite, a considerable amount of heterocharges and homocharges accumulate near cathode and inside the samples, respectively. Whereas, only a small number of heterocharges arise near anode in the 1.5wt%TAIC-*s*-SiO_2_/XLPE and 2.0wt%TAIC-*s*-SiO_2_/XLPE nanocomposites, as shown in Figure 9b,c, which imply that homocharge accumulation can be inhibited by increasing the concentration of TAIC-*s*-SiO_2_ nanofillers.

**Figure 10:**
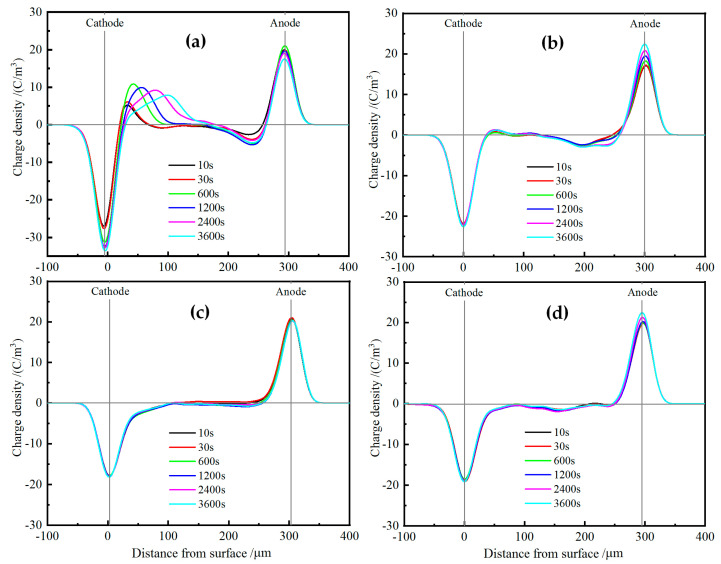
Space charge distributions in (**a**) pure XLPE and the nanocomposites of (**b**) 0.5wt%TAIC-*s*-SiO_2_/XLPE, (**c**) 1.5wt%TAIC-*s*-SiO_2_/XLPE and (**d**) 2.0wt%TAIC-*s*-SiO_2_/XLPE under DC electric field of 40 kV/mm at 60 °C.

When the temperature is further raised to 60 °C, the injection depth of the evident heterocharges near both electrodes in XLPE apparently increase with the polarization time, as shown in Figure 10a. The homocharge injection inside the 0.5wt%TAIC-*s*-SiO_2_/XLPE nanocomposite alternates to the heterocharge accumulation when the temperature is raised from 40 °C to 60 °C, and a substantial homocharge injection begin to arise near cathode for the other two nanocomposites with higher TAIC-*s*-SiO_2_ concentrations, as shown in Figure 10b,c. It is also noted that the negative space charges have been injected from anode into the internal of 2.0wt%TAIC-*s*-SiO_2_/XLPE nanocomposite when the temperature approaches 60 °C.

**Figure 11:**
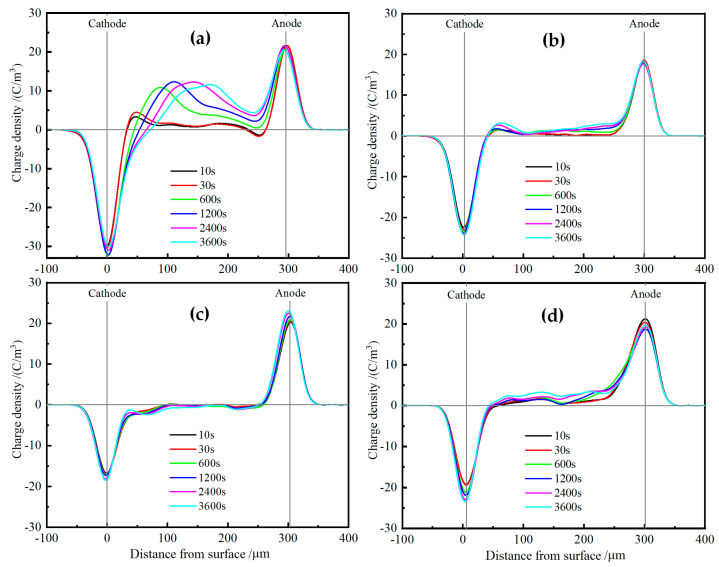
Space charge distributions in (**a**) pure XLPE and the nanocomposites of (**b**) 0.5wt%TAIC-*s*-SiO_2_/XLPE, (**c**) 1.5wt%TAIC-*s*-SiO_2_/XLPE and (**d**) 2.0wt%TAIC-*s*-SiO_2_/XLPE under DC electric field of 40 kV/mm at 80 °C.

At the temperature of 80 °C, large numbers of heterocharges accumulate near cathode and inject through the XLPE samples as the polarization time is prolonged, as shown in Figure 11a. In comparison, only a small number of heterocharges and homocharges accumulate at cathode in 0.5wt%TAIC-*s*-SiO_2_/XLPE and 1.5wt%TAIC-*s*-SiO_2_/XLPE nanocomposites respectively, while significant positive charges have been injected from anode into the material interior of 2.0wt%TAIC-*s*-SiO_2_/XLPE nanocomposite, as shown in Figure 11b,c. This result is a further manifestation that filling TAIC-*s*-SiO_2_ and increasing its concentration can impede the hetercharge injections.

**Figure 12:**
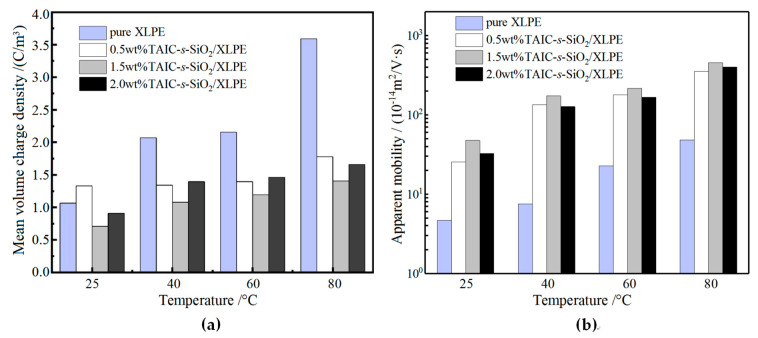
(**a**) Mean volume density of space charges under DC electric field of 40 kV/mm for 3600 s polarization time; (**b**) apparent mobility of space charges at the beginning of depolarization progress in short circuit after 3600 s polarization.

In order to further quantitatively analyze space charge accumulations in the prepared nanocomposites with modified dielectric performances at elevated temperatures, the average space charge densities under 40 kV/mm electric field after polarizing for 3600 s are calculated according to the formula defined as follows [35,36]:

(3) $$q(t,E_{p})=\frac{1}{x_{1}-x_{0}}\int_{x_{0}}^{x_{1}}\left|q_{p}(x,t,E_{p})\right|dx$$

where *q*_p_(*x*, *t*, *E*_P_) denotes the internal space charge density at position *x*; *t* represents polarization time; *E*_P_ symbolizes the applied electric field magnitude; *x*_0_ and *x*_1_ identify the interior positions at the down and up electrodes, respectively. It is indicated from the calculation results shown in Figure 12a that the average charge density of all samples except 0.5wt%TAIC-*s*-SiO_2_/XLPE nanocomposite increases with the increasing temperature, confirming that temperature elevation will exacerbate space charge injections from both electrodes, which is mostly intensified in pure XLPE. In particular, it is found that 1.5wt%TAIC-*s*-SiO_2_/XLPE nanocomposite represents the lowest average charge density throughout the investigated temperature range.

The improvement in the space charge characteristics of polymers by filling nanoparticles is generally attributed to the introduction of deep traps at the interfaces between nanofillers and polymer matrix, which will restrict charge transports and reduce charge carrier mobility, thereby leading to the abatement of space charge accumulations. It is assumed that the recombination of space charges and the mean free time of charge migrations relative to the residence time of charges in deep traps are both negligible, so the apparent charge mobility can be approximately estimated by the decaying characteristics of space charges [37]. The electrical mobility of space charges can be estimated according to the change curves of mean volume charge density with time at different temperatures by the following equation:

(4) $$\mu (t)=\frac{\varepsilon_{0}\varepsilon_{r}}{q^{2}(t)}\cdot \frac{dq(t)}{dt}$$

where d*q*(*t*)/d*t* and *q*(*t*) denote the decaying rate and instantaneous value of mean volume charge density after short-circuit, respectively; and *ε*_r_ and *ε*_0_ are the relative and vacuum permittivities, respectively. According to Equation (4), the apparent mobility of pure XLPE and XLPE nanocomposites at various temperatures can be calculated from the depolarization characteristic curves of space charges, which represents the migration rate of the charges captured in traps. Figure 12b shows the calculated apparent mobility at the beginning of depolarization in short-circuit after polarizing for 3600 s (polarization time of space charge accumulation). For the pure XLPE and all the composite materials, the apparent mobility of space charges increases gradually with the increase of temperature. In comparison, the 1.5wt%TAIC-*s*-SiO_2_/XLPE composite shows the highest apparent mobility of space charges in the measured temperature range, which is consistent with the mean volume charge density of space charges shown in Figure 12a. It is thus proved that the increase of current carrier mobility will lead to the suppression of space charge accumulations.

**Figure 13:**
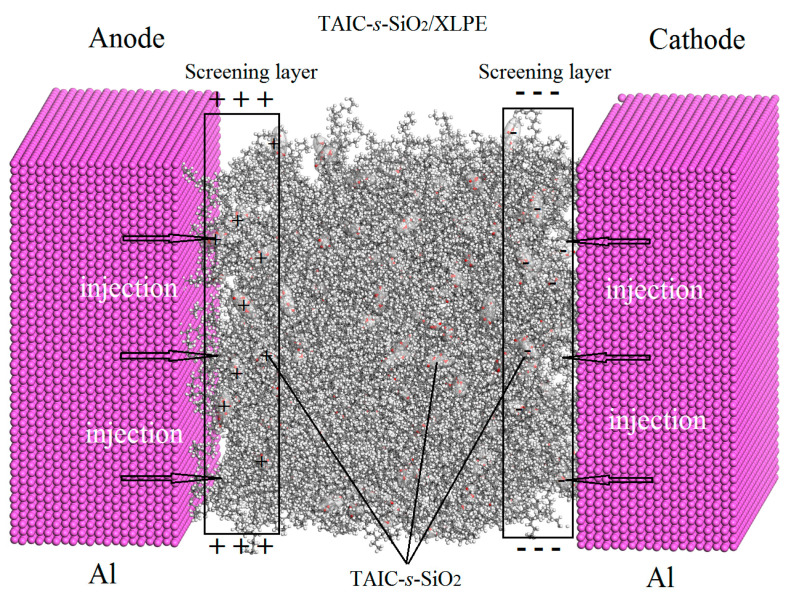
Schematic Coulomb potential shielding mechanism for suppressing the space charge accumulations in TAIC-*s*-SiO_2_/XLPE nanocomposites under DC electric field.

In brief, the specific nanosurface properties and the small size effects of inorganic nanofillers can render deep level charge traps to inhibit carrier transport and suppress space charge injections by forming the charge shielding layers near electrodes [38,39]. These efficient deep traps mainly originate from the polar-groups distributing at the interfaces between the polymer matrix and inorganic nano-additives. The polar-groups of auxiliary crosslinkers grafted onto the nanosilica surfaces are bound to have a significant effect on the spatial distribution and energy level of the charge traps. The uniformly distributed deep traps introduced by polar-groups of TAIC molecules fixed on nanosilica surfaces will effectively capture the injected heterocharges and thus form Coulomb potential screening layers near electrodes under DC electric field, which can impede further charge injections from electrodes, as schematically shown in Figure 13.

## 4. Conclusions

In the high interest of simultaneously ameliorating the crosslinking degree, space charge characteristics and dielectric breakdown strength of the SiO_2_/UV-XLPE nanocomposites prepared with UV-initiated polyethylene crosslinking and nanodielectric technologies, the chemical functionalization of grafting auxiliary crosslinker onto the surface of SiO_2_ nanoparticles is employed for comprehensive modifications. FTIR and ^1^H-NMR tests verify that auxiliary crosslinker TAIC has been successfully grafted onto the surface of SiO_2_ nanoparticles. The TAIC molecules on the surface of SiO_2_ nanoparticles and the macro-molecular photon-initiator will not be evaporated in the crosslinking reaction, resulting in a significant increment of crosslinking degree. Gel content of SiO_2_/UV-XLPE nanocomposite can be further raised by increasing the concentration of TAIC-*s*-SiO_2_ nanofillers when the filling content is <1.5 wt%, which is attributed to the more UV absorption on the surface of silica nanofillers with a higher dispersivity and larger specific surface areas. When the TAIC-*s*-SiO_2_ concentration is increased to 2 wt%, the silica nanofillers aggregate into larger particles with a reduced specific surface area in polyethylene matrix, accounting for the abatement in UV absorption and auxiliary crosslinking efficiency. Compared with the UV-XLPE insulating material, the SiO_2_/UV-XLPE nanocomposites acquire significantly improved dielectric breakdown strength and space charge characteristics by exploiting the SiO_2_ nanofillers with the surface functionalized by TAIC molecules containing polar-groups, especially at elevated temperatures approaching 80 °C. The 1.5wt%TAIC-*s*-SiO_2_/XLPE nanocomposite represents the lowest average density of space charges throughout the investigated range of temperatures. The polar-group in the TAIC molecules grafted on the surface of silica nanofillers can present a high density of effective charge traps and form a Coulomb potential screening layer near electrode to improve the dielectric breakdown strength and space charge characteristics. By utilizing the functionalized nanosilica with an appropriately increased filling concentration, the crosslinking efficiency of polyethylene molecules can be definitely promoted simultaneously with a remarkable suppression on the heterocharge accumulations in UV-XLPE. This paper suggests a prospective method to develop high performance UV-XLPE insulating materials.

## Figures and Tables

**Table 1:** Mixture components for preparing UV-XLPE nanocomposites.

Sample	LLDPE/wt%	BPL/wt%	TAIC/wt%	TAIC-*s*-SiO_2_/wt%	Irganox1010/wt%
XLPE	96.7	2	1	0	0.3
0.5 wt%TAIC-*s*-SiO_2_/XLPE	97.2	2	0	0.5	0.3
1.5 wt%TAIC-*s*-SiO_2_/XLPE	96.7	2	0	1	0.3
2.0 wt%TAIC-*s*-SiO_2_/XLPE	95.7	2	0	2	0.3

**Table 2:** Characteristic breakdown field *E*_b_ at 63.2% probability and shape parameter *β* obtained by fitting the DBS experimental data with 2-parameter Weibull distribution at 95% confidence interval.

Materials	2-Parameter									
	*E*_b_/(kV/mm)					*β*				
	25 °C	40 °C	60 °C	80 °C	100 °C	25 °C	40 °C	60 °C	80 °C	100 °C
XLPE	334.4	307.8	252.9	229.2	170.7	6.27	10.59	11.77	13.30	14.13
0.5wt%TAIC-*s*-SiO_2_/XLPE	352.2	307.7	254.5	214.4	162.0	5.46	7.99	12.03	14.71	15.75
1.5wt%TAIC-*s*-SiO_2_/XLPE	366.4	328.6	260.1	235.1	170.5	5.92	7.24	13.78	13.00	14.29
2.0wt%TAIC-*s*-SiO_2_/XLPE	359.1	295.2	249.4	222.9	159.6	5.81	9.45	10.25	15.71	16.76
